# Mean Duration for Cessation of Pain following Tonsillectomy Operation among the Patients of Otolaryngology Department in a Tertiary Care Hospital: A Descriptive Cross-sectional Study

**DOI:** 10.31729/jnma.7120

**Published:** 2021-10-31

**Authors:** Nain Bahadur Mahato, Meera Bista, Bhuwan Bhandari, Anil Maharjan

**Affiliations:** 1 Department of ENT-HNS, Kathmandu Medical College, Sinamangal, Kathmandu, Nepal; 2 Department of Surgery, Nepal National Hospital, Kalanki, Kathmandu, Nepal; 3 Department of Anesthesia and Critical Care, Kist Medical Collge, Imadol, Lalitpur, Nepal

**Keywords:** *analgesia*, *pain*, *tonsillectomy*

## Abstract

**Introduction:**

Tonsillectomy is one of the most common surgical procedures performed by Otolaryngologists world-wide. There are various techniques for tonsillectomy, but none of the techniques has been accepted as the best one universally. Despite the efforts of all the surgeon and use of recent techniques, some post-tonsillectomy morbidity is unavoidable. The main objective of our study is to find out the mean duration for cessation of pain among the patients following tonsillectomy operation in a tertiary care hospital.

**Methods:**

This is a descriptive cross-sectional study of 104 patients who underwent tonsillectomy in department of otolaryngology of Kathmandu Medical College from 1^st^ August 2020 to 31^st^ July 2021. Convenient sampling technique was used. Ethical Approval was taken from Ethical Clearance Committee of hospital (Reference number: 2207202005). Proforma containing visual analog score was given to every patient for scoring the severity of postoperative pain. The scoring of pain was done from 1st postoperative day till 14^th^ postoperative day. Descriptive statistical analysis was done.

**Results:**

One hundred four patients had undergone tonsillectomy in our hospital. The mean duration for cessation of pain was 10 (9.75+1.97) days with mean duration of analgesia taken of 11 (10.84+2.15) days. The mean duration after tonsillectomy operation for cessation of pain on drinking was 8 (7.51±1.19) days and on eating solids 12 (11.59±2.56) days. Patients reported the first normal night of sleep at seven (6.90+1.41) days and return to normal daily activities 11 (11.18+2.53) days.

**Conclusions:**

From the study concluded that the mean duration for cessation of pain after tonsillectomy is slightly lower than other similar study.

## INTRODUCTION

Tonsillectomy is the oldest surgical procedures performed by Otolaryngologists world-wide for the various indications.^1^ Tonsillectomy is performed by a variety of techniques.^2^ These techniques have evolved over the years aiming to make the procedure safe, decrease the surgical time, intra operative blood loss, postoperative morbidity, and complications.^3^ Despite the different techniques available for tonsillectomy, none of the techniques has been accepted as the best one universally.^4-6^

Postoperative pain cessation varies during different day-today activities. Reactionary hemorrhage is the most feared complication post-tonsillectomy, despite the efforts of all surgeons to low reactionary hemorrhage rates seem to be unavoidable. Incidence of haemorrhage is not related to grade and seniority of the surgeon.^7^ Cold steel dissection has higher incidence of reactionary haemorrhage and diathermy has greater incidence of secondary haemorrhage.^8^

The main objective of our study is to find out the mean duration for cessation of pain among the patients following tonsillectomy operation in a tertiary care hospital.

## METHODS

This is a descriptive cross-sectional study of 104 patients who underwent tonsillectomy in department of otolaryngology of Kathmandu Medical College over a period of 1 year duration from 1st August 2020 to 31st July 2021. Ethical Approval was taken from Ethical Clearance Committee of KMCTH(Reference number: 2207202005). Patients with history of recurrent tonsillitis, chronic tonsillitis, obstructive sleep apnoea syndrome (OSAS), second attack of quinsy were included in our study. The patients with bleeding disorders, hemoglobin level <10gm%, severe trismus, any chronic illness affecting recovery were excluded from the study. Convenient sampling technique was used.

The sample size was calculated by using the formula,

n = Z^2^ × σ^2^ / e^2^

  = (1.96)^2^ × (0.5)^2^ / (0.1)^2^

  = 96

Where,

n= required sample size
Z = 1.96 at 95% Confidence Interval (CI)
σ = standard deviation for mean duration of cessation of pain among the patients following tonsillectomy operation, 0.5
e = margin of error, 10%

Here, the calculated sample size is 96, taking nonresponse rate 8%, the sample size is 104. All the patients to be enrolled in our study were examined by consultant ENT surgeon. History regarding the indications of tonsillectomy and mouth opening were evaluated. Anterior rhinoscopy was done to find out deviated nasal septum (DNS) which would have obscured nasal intubation. All the patients undergoing tonsillectomy were positioned in Rose's position and anesthetized by using nasal Ring-Adair-Elwin (RAE) endotracheal tube. The patients' mouth was held open with Boyle-Davis gag for adequate exposure of the oropharynx. The patients were operated by two different techniques, Cold Dissection Technique (CDT) and Bipolar Electrocautery Technique (BET). The average time taken in both the procedures was 45 minutes.

In Cold Dissection Technique, tonsil was grasped with tonsil holding forceps and the anterior tonsillar pillar was cut with No. 12 knife. Surgical plane between the tonsillar capsule and superior constrictor muscle was identified and was dissected from the superior pole towards the lower pole by a serrated tonsil dissector. Inferior pole pedicle was crushed and cut completely by using Eve's tonsillar snare. Following dissection, the tonsillar fossa was packed with a cotton swab for a few minutes and then, the other tonsil was similarly removed. Finally, the gauzes were removed and when necessary, bipolar electrocautery was used to secure hemostasis.

In Bipolar Electrocautery Technique, bipolar electrocautery forceps, set at a power of 35-40 W was used. In this method, incision on the anterior tonsillar pillar was made from superior pole towards inferior pole using the tip of a bipolar forceps. Superior pole of the tonsil was dissected off from the tonsillar fossa in the surgical plane. During dissection, encountered vessels were cauterized and then separated from the tonsil. The inferior pedicle was cauterized and the tonsil was completely removed. Any further hemostasis of the tonsillar fossa was secured by coagulation with the bipolar forceps. After securing complete hemostasis of both the tonsillar fossa, the gauzes and throat pack were removed. After extubation, post-tonsillectomy position (left lateral) was maintained in the postoperative ward for about 6 hours.

The patients were treated with intravenous 3rd generation cephalosporins and supportive medications for at least 5 days in the hospital. Proforma containing visual analog score was given to every patient for scoring the severity of postoperative pain. The scoring of pain was done from 1st postoperative day till 14th postoperative day. The data for mean duration for cessation of pain on drinking, on eating of solids, first normal night of sleep, return to normal daily activities were collected and analyzed for two different techniques. Descriptive statistical analysis was done.

## RESULTS

One hundred four patients, age ranging from 15-50 years were enrolled in our study. The mean duration for cessation of pain was 10 (9.75±1.97) days after tonsillectomy. The mean duration of analgesia taken of 11 (10.84 ±2.15) days. The mean duration for cessation of pain on drinking was 8 (7.51±1.19) days and on eating solids 12 (11.59±2.56) days. Patients reported the first normal night of sleep at seven (6.90±1.41) days and return to normal daily activities 11 (11.18±2.53) days (Table 1).

**Table 1:** Mean duration for cessation of pain during different activities.

Variables	Minimum	Maximum	Mean±SD
Mean duration for cessation of pain (3-21) days	6	15	9.75±1.979
Mean duration for cessation of pain on drinking (1-18) days	5	10	7.51±1.199
Mean duration for cessation of pain on eating solids (1-20) days	7	18	11.59±2.560
Mean duration of analgesic taken (5-25) days	7	17	10.84±2.155
First normal night of sleep (0-18) days	4	10	6.90±1.418
Return to normal daily activities 12 (2-24) days	7	19	11.18±2.530

Reactionary hemorrhage was seen in 3 (2.88%) patients, while secondary hemorrhage was seen in 2 (1.92%) patients. Postoperative fever was seen in 5 (4.80%) patients, 27 (25.96%) patients had referred otalgia and oedema of uvula was seen in 12 (11.53%) patients (Table 2).

**Table 2:** Number of patients with postoperative morbidity after tonsillectomy.

Postoperative Morbidity	Post-Tonsillectomy n (%)
Reactionary Hemorrhage	3 (2.88)
Secondary Hemorrhage	2 (1.92)
Postoperative Fever	5 (4.80)
Otalgia	27 (25.96)
Oedema of Uvula	12 (11.53)

Among the total patients, the tonsillectomy was done by cold dissection technique in 44 (42.30%) and 60 (57.69%) by bipolar electro cautery technique.

The mean duration for cessation of pain was 9 (9.07±2.00) days with mean duration of analgesia taken of 11 (10.57±2.12) days in CDT (Table 3).

**Table 3:** Mean duration for cessation of pain during cold dissection technique.

Variables	Mean±SD
Mean duration for cessation of pain (3-21) days	9.07±2.005
Mean duration for cessation of pain on drinking (1-18) days	6.80±1.250
Mean duration for cessation of pain on eating solids (1-20) days	10.61±2.517
Median duration of analgesic taken (5-25) days	10.57±2.128
First normal night of sleep (0-18) days	6.59±1.419
Return to normal daily activities 12 (2-24) days	11.07±2.848

The mean duration for cessation of pain was 10 (10.25±1.81) days in BET group with mean duration of analgesia taken of 11 (11.03 ±2.17) days in BET group (Table 4).

**Table 4:** Mean duration for cessation of pain during electrocautery technique.

Variables	Mean±SD
Mean duration for cessation of pain (3-21) days	10.25±1.819
Mean duration for cessation of pain on drinking (1-18) days	8.03 ± 0.843
Mean duration for cessation of pain on eating solids (1-20) days	12.30±2.367
Mean duration of analgesic taken (5-25) days	11.03±2.170
First normal night of sleep (0-18) days	7.13±1.384
Return to normal daily activities 12 (2-24) days	11.27±2.291

## DISCUSSION

History of tonsillectomy dates back to 3000 years ago with the first report referring to Hindu medicine about 1000 years B.C. In 40 AD, a Roman surgeon, Cornelius Celsus performed this operation for the first time using his fingernails.^9^ He also described scraping the tonsils and cutting them out by a hook-like instrument.^10^ At the beginning of the twentieth century that Worthington^11^ described the modern technique of tonsillectomy by dissection. In 1909, a surgeon named Cohen adopted ligature of bleeding vessels to control per operative bleeding and thereafter, tonsillectomy became a common and safe procedure in hospitals around the world.^10^ In 1968, Remington-Hobbs, Haase and Noguera^12^ in 1969 described the use of diathermy for removal of tonsils.^13^ In 1982 Goycolea described electrodissection by using monopolar diathermy^14^ and Pang, 10 years later, reported the first tonsillectomy by bipolar electrocautery.^2^

Nowadays, tonsillectomy is performed with a variety of techniques such as: conventional cold dissection, mono and bipolar electrocauteries, cryosurgery, harmonic, coblation, radiofrequency and laser. All these techniques have advantages as well as drawbacks, as reported by the surgeons from time to time, hence none of them have been accepted as the single best technique universally. Over the last century many different techniques of tonsillectomy have been described, of which Cold Dissection-Snare method and Bipolar Electro dissection methods are commonly used. These methods have frequently been compared with each other by different investigators around the world, addressing especially the conventional cold dissection technique (CDT) versus the bipolar electrocautery technique (BET). In our study, we decided to assess the mean duration for cessation of postoperative pain after tonsillectomy during different day-today activities.

We found the mean duration for cessation of pain was 10 days (range, 3-21 days) with mean duration of analgesia taken of 11 days (range, 5-25 days) which is slightly lower than the previous study. ^15^ More than 52% of the patients needed 1 to 3 rescue analgesic doses daily during the first week after tonsillectomy. The mean duration for cessation of pain on drinking was 8 days (range, 1-18 days) and on eating solids 12 days (range, 1-20 days). Patients reported the first normal night of sleep at seven days (range, 0-18 days) and return to normal daily activities at 11 days (range, 2-24 days). Cardozo AA and colleagues have noted positive relationship between the total amount of bipolar diathermy used and postoperative pain.^16^ Similarly, Atallah, et al.^17^ found an increase in pain and a related prolongation of oral intake time in the postoperative period done by using bipolar cautery tonsillectomy. Increase in postoperative pain in intensity and duration in bipolar diathermy cases; was also observed by Fiona, et al.^18^

In our study, 3 (2.88%) patients had reactionary hemorrhage, for which conservative treatment was sufficient to control the bleeding. Two (1.92%) patients, had secondary hemorrhage on 2nd week for which 1 patient required operation theatre for bleeding control and the other patient was managed conservatively in the ward. Pang YT has reported incidence of postoperative hemorrhage as 1.7% with bipolar diathermy tonsillectomy compared to 3.4% with cold dissection.^2^ Stephen O'Leary, et al. in his study reported that the difference in the risk of bleeding after dissection and diathermy tonsillectomy did not reach statistical significance.^8^ Whereas Gendy S and colleagues have reported a higher incidence of secondary haemorrhage with bipolar dissection (2.3%) compared to cold dissection (1%) which is similar to our study.^3^ One explanation for higher post-tonsillectomy bleeding rates after diathermy techniques may be related to greater thermal damage as the result of excessively high power settings or excessively frequent or prolonged application of diathermy.^19^

Referred otalgia after tonsillectomy is one of the common postoperative morbidity. Twenty seven (25.96%) patients in our study complained of referred otalgia following tonsillectomy. Study done by Kurt Breson and Jeep Diepeveen^20^ (28%), Niels Rasmussen^21^ (25.9%), Al-Yasiri (25%), Roos K, Lind L (32%) had similar results of referred otalgia as our study. Oedema of uvula was seen in 12 (11.53%) patients, this is normal and is due to the cauterization of the tonsil blood vessels that forces the uvula to swell up until the glands develop an alternative drainage pattern.

Postoperative fever is a common problem after tonsillectomy; 3 (2.88%) patients having a temperature higher than 37.5°C and 2 (1.92%) patients higher than 38°C in the first 24 hours post-tonsillectomy. Fever may occur due to trauma associated surgery that elicits the production of pyrogenic cytokines in the absence of infection. The production of host endogenous pyrogens, or pyrogenic cytokines is the final common pathway through which fever occurs from such diverse causes as infection and trauma in the absence of infection. Pyrogenic cytokines have various effects on stimulating or controlling the inflammatory response and fever. Cytokines act as an intracellular mediator and messenger controlling the host response to injury and infection.^22^

## CONCLUSIONS

The mean duration for cessation of pain after tonsillectomy is slightly lower than other similar study. Despite the different techniques and efforts of all the surgeons, some postoperative morbidity is unavoidable. Reactionary hemorrhage is the most feared complication post-tonsillectomy because of the risk of airway obstruction, shock and ultimately death if inappropriately managed. Excessive pain associated with referred otalgia, postoperative fever, oedema of the uvula, infection in tonsillar fossa promoting secondary hemorrhage etc are the other complications mainly seen in our practice.
